# Predictors of successful discontinuation of continuous kidney replacement therapy in a pediatric cohort

**DOI:** 10.1007/s00467-022-05782-0

**Published:** 2022-10-31

**Authors:** Elizabeth Y. Wei, Kim T. Vuong, Euyhyun Lee, Lin Liu, Elizabeth Ingulli, Nicole G. Coufal

**Affiliations:** 1 Department of Pediatrics, Division of Critical Care, University of California San Diego, 3020 Children’s Way, MC 5065, San Diego, CA 92123 USA; 2 Rady Children’s Hospital, San Diego, CA USA; 3 Department of Pediatrics, University of California San Diego, San Diego, CA USA; 4 Altman Clinical and Translational Research Institute, University of California San Diego, San Diego, CA USA; 5 Division of Biostatistics and Bioinformatics, Herbert Wertheim School of Public Health and Human Longevity Science, University of California San Diego, San Diego, CA USA; 6 Department of Pediatrics, Division of Nephrology, University of California San Diego, San Diego, CA USA

**Keywords:** Continuous kidney replacement therapy, Time factors, Acute kidney injury–therapy, Acute kidney injury–rehabilitation, Pediatric intensive care units, Extracorporeal dialysis

## Abstract

**Background:**

Recognizing the optimal time to discontinue continuous kidney replacement therapy (CKRT) is necessary to advance patient recovery and mitigate complications. The aim of this study was to identify predictors of successful CKRT cessation in pediatric patients.

**Methods:**

All patients requiring CKRT between January 2010 and March 2021 were evaluated. Patients on peritoneal or hemodialysis, who transferred between institutions, or who did not trial off CKRT were excluded. Successful discontinuation was defined as remaining off CKRT for at least 7 days. Demographics, admission diagnoses, PRISM III scores, and reasons for CKRT initiation were obtained. Clinical and biochemical variables were evaluated at CKRT initiation and discontinuation and in the 12-h period following discontinuation. Comparisons were conducted using Wilcoxon rank sum and Fisher’s exact tests for continuous and categorical variables, respectively. A logistic regression model was fitted to identify significant factors.

**Results:**

Ninety-nine patients underwent a trial off CKRT. Admission and initiation characteristics of the success and failure groups were similar. Patients who required re-initiation (*n* = 26) had longer ICU lengths of stay (27.2 vs. 44.5 days, *p* = 0.046) and higher in-hospital mortality (15.1% vs. 46.2%, *p* = 0.002). Urine output greater than 0.5 mL/kg/h irrespective of diuretic administration in the 6-h period before CKRT discontinuation was a significant predictor (AUC 0.72, 95% CI 0.60–0.84, *p* = 0.0009).

**Conclusions:**

Determining the predictors of sustained CKRT discontinuation is critical. Urine output greater than 0.5 mL/kg/h in this pediatric cohort predicted successful discontinuation. Future studies are needed to validate this threshold in disease- and age-specific cohorts and evaluate additional biomarkers of kidney injury.

**Graphical abstract:**

**Figure:**
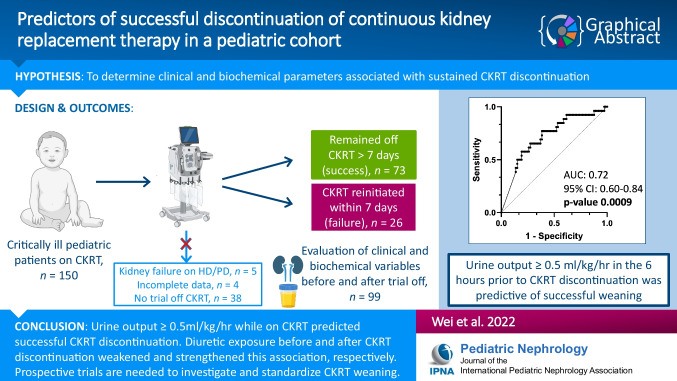
A higher resolution version of the Graphical abstract is available as Supplementary information

**Supplementary Information:**

The online version contains supplementary material available at 10.1007/s00467-022-05782-0.

## Introduction

Continuous kidney replacement therapy (CKRT) is employed in 1.5% of hospitalized children with severe acute kidney injury (AKI) defined as Kidney Disease: Improving Global Outcomes (KDIGO) stage 2 or 3 [1]. Mortality during CKRT ranges between 39 and 44.7% and is associated with longer duration of CKRT, higher percentage fluid overload (% FO), higher blood urea nitrogen (BUN), higher illness severity scores, sepsis and multiple organ dysfunction syndrome (MODS), and need for mechanical ventilation, extracorporeal membrane oxygenation (ECMO), bone marrow transplant, or cardiopulmonary bypass [2–6].

Although CKRT has become safer with advancements in hemofilter biocompatibility, pediatric-appropriate circuits, and machine technologies, complications such as bleeding, thrombosis, infection, and hemodynamic instability still occur [7]. Furthermore, CKRT also impacts a patient’s nutritional status [8, 9], interferes with drug delivery and clearance [10], and is resource intensive [11]. There is data to suggest that timing of CKRT initiation may impact outcomes in both adult and pediatric patients, but there currently exists a paucity of knowledge regarding appropriate timing for pediatric CKRT discontinuation [2, 12–14].

Our objective was to describe clinical and biochemical factors associated with successful CKRT discontinuation in critically ill pediatric patients.

## Patients and methods

### Study population and setting

This single center retrospective cohort study in a tertiary pediatric referral center included all patients in our neonatal, pediatric, and cardiothoracic intensive care units who received CKRT from January 1, 2010, to March 15, 2021. Patients with a previous diagnosis of kidney failure on peritoneal dialysis or hemodialysis, those transferred from other institutions with deficient clinical data, those who received slow continuous ultrafiltration (SCUF) alone without dialysis, and those who never had a trial off CKRT due to death or withdrawal of life-sustaining therapies were excluded. All patients initiated on CKRT at our institution received continuous venovenous hemodiafiltration with regional citrate anticoagulation. Patients with indications for systemic anticoagulation, such as venous thromboembolism or extracorporeal membrane oxygenation, received heparin. No patients received bivalirudin during the study period.

Trial off CKRT was at the discretion of the care team and therefore reflected individual variations in practice. Patients were divided into those who successfully remained off CKRT for at least 7 days (success group) and those who required re-initiation of CKRT within this period (failure group). Any period off CKRT for 12 h or more was considered a trial off. Subsequent use of peritoneal dialysis or intermittent hemodialysis was not considered failure of CKRT discontinuation.

### Data collection

Patient demographic data (age, weight, and gender), admission diagnoses, comorbid conditions, illness severity scores (pediatric risk of mortality score [PRISM] III), ICU and hospital lengths of stay (LOS), and in-hospital mortality were obtained for all patients. Clinical and biochemical data were extracted from the medical record upon admission to the ICU, at CKRT initiation, at CKRT discontinuation, and after CKRT discontinuation. Clinical data included mechanical ventilation, mean arterial pressures (MAP) and central venous pressures (CVP), vasoactive inotropic score (VIS), fenoldopam administration, diuretic administration, percentage fluid overload (% FO), and urine output (UO). Biochemical variables collected included pH, PaCO2, lactate, BUN, serum creatinine, sodium, potassium, bicarbonate, albumin, white blood cell count, hemoglobin, platelet count, C-reactive protein (CRP), and international normalized ratio (INR).

UO was collected 6 h prior to CKRT initiation, 6 and 24 h prior to CKRT discontinuation, and 6 and 12 h after CKRT discontinuation.

The VIS was calculated using the formula: 1 × (dopamine + dobutamine [µg/kg/min]) + 10 × milrinone (µg/kg/min) + 100 × (epinephrine + norepinephrine [µg/kg/min]) + 10,000 × vasopressin (U/kg/h) [15].

To appropriately compare diuretic administration among patients, loop diuretic doses were converted into furosemide equivalents per kilogram (FE/kg) with 1 mg bumetanide = 40 mg furosemide for intravenous diuretics [16]. The maximum dose of continuous bumetanide or furosemide infusions was used in the calculations.

The % FO was defined using the formula conceived by Goldstein et al.: (total fluid in – total fluid out in liters)/(ICU admission weight in kilograms) × 100% [4, 17]. Change in % FO during the CKRT course was obtained.

### Statistical analysis

Medians with interquartile range (IQR 25th and 75th percentiles) and frequency with percentages were used to describe continuous and categorical variables, respectively. Comparisons between the two outcome groups were made using Wilcoxon rank sum and Fisher’s exact tests.

A univariable logistic regression was fitted using urine output at multiple time periods against the CKRT outcome. This model was used to plot a receiver operating characteristic (ROC) curve and to calculate area under the curve (AUC). The optimal urine output thresholds were determined using Youden’s index, and the corresponding sensitivity and specificity were estimated.

To identify independent predictors of CKRT discontinuation, univariable logistic regression was first conducted to examine the association between CKRT discontinuation and a priori selected covariates, i.e., CKRT duration, BUN at CKRT initiation, cardiopulmonary bypass, pre-existing cardiac disease, CKRT utilization for sepsis, and UO in the 6 h prior to discontinuation. A *p* value < 0.2 from the univariable logistic regression was required for entry into the multivariable model. A backward elimination of non-significant predictors from the model was performed; variables with the highest *p* values were removed sequentially until all predictors in the multivariable model had a *p* value < 0.1. A two-tailed *p* value less than 0.05 was considered statistically significant. All analyses were performed using R Version 4.1.2 (R Foundation for Statistical Computing, Vienna, Austria. http://www.R-project.org/).

This study was approved by the University of California, San Diego Institutional Research Ethics Committee with a waiver of informed consent.

## Results

### Patient characteristics

**Fig. 1:**
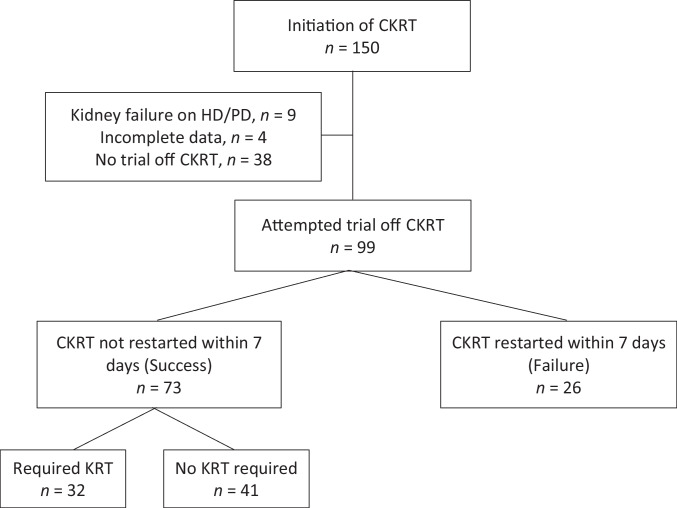
All patients initiated on continuous kidney replacement therapy (CKRT) from 1/1/2021 to 3/15/2021 screened for study inclusion. Of the patients who required kidney replacement therapy (KRT) after successful discontinuation, four patients were discharged with new dialysis dependence

During the study period, 150 patients received CKRT (Fig. 1). Fifty-one patients were excluded: nine carried a previous diagnosis of kidney failure, four had incomplete data, and 38 died or had withdrawal of life-sustaining therapies. Of the remaining 99 patients, 73 patients remained off CKRT for greater than 7 days (success), and 26 required CKRT re-initiation (failure).

The median patient age was 6.4 years (IQR 1.6–14.0) with a median admission weight of 23.5 kg (IQR 10.9–45.6). Slightly more than half (51.5%) of the cohort was female (Table 1).

**Table 1:** Demographics of study patients who successfully remained off continuous kidney replacement therapy and those who required re-initiation within 7 days

	CKRT outcome		
	Success *n* = 73	Failure *n* = 26	*p* value
Age (year)†	6.3 (2.0–13.0)	8.8 (1.1–15.9)	0.494
Female, *n* (%)	37 (50.7)	14 (53.8)	0.823
Admission weight (kilograms)†	24.0 (11.3–40.0)	20.8 (7.3–48.3)	0.930
**Pre-existing kidney disease**			
None, *n* (%)	68 (93.2)	21 (80.8)	0.122
Chronic kidney disease, *n* (%)	2 (2.7)	2 (7.7)	0.281
Other, *n* (%)	3 (4.1)	3 (11.5)	0.184
**Pre-morbid conditions**			
Cardiac disease, *n* (%)	14 (19.2)	10 (38.5)	0.063
Liver disease, *n* (%)	8 (11.0)	3 (11.5)	1.000
PRISM III score†	12 (7–20)	13 (10–17)	0.839
**Diagnostic group**			
Post-operative, *n* (%)	8 (11.0)	5 (19.2)	0.317
Post-op cardiopulmonary bypass, *n* (%)	4 (5.5)	4 (15.4)	0.201
Cardiovascular, *n* (%)	19 (26.0)	10 (38.5)	0.315
Respiratory, *n* (%)	17 (23.3)	8 (30.8)	0.444
Neurologic, *n* (%)	12 (16.4)	2 (7.7)	0.344
Gastrointestinal, *n* (%)	15 (20.5)	4 (15.4)	0.773
Sepsis, *n* (%)	42 (57.5)	12 (46.2)	0.364
Oncologic, *n* (%)	10 (13.7)	8 (30.8)	0.075
Other, *n* (%)	13 (17.8)	4 (15.4)	1.000
**Reason for CKRT initiation**			
Fluid overload, *n* (%)	47 (64.4)	18 (69.2)	0.811
Anuria/oliguria, *n* (%)	36 (49.3)	14 (53.8)	0.820
Electrolyte abnormality, *n* (%)	13 (18.1)	4 (16.0)	1.000
Uremia, *n* (%)	17 (23.3)	9 (34.6)	0.303
Acidosis, *n* (%)	19 (26.0)	3 (11.5)	0.172
Sepsis, *n* (%)	21 (28.8)	4 (15.4)	0.202
Hyperammonemia, *n* (%)	6 (8.2)	1 (3.8)	0.672
Other, *n* (%)	8 (11.0)	2 (7.7)	1.000
CKRT duration (hours)†	171 (62–258)	114 (58–238)	0.462
CKRT on ECMO, *n* (%)	15 (20.5)	6 (23.1)	0.785
**Peritoneal or hemodialysis use**			
Prior to CKRT, *n* (%)	8 (11.0)	3 (11.5)	1.000
After CKRT, *n* (%)	32 (43.8)	9 (34.6)	0.490
Discharged on dialysis, *n* (%)	4 (12.5)	2 (22.2)	0.326
ICU length of stay (days)†	27.2 (12.9–53.8)	44.5 (29.7–67.9)	0.046^*^
Hospital length of stay (days)†	38.0 (24.0–75.0)	49.0 (37.0–81.8)	0.246
In-hospital mortality, *n* (%)	11 (15.1)	12 (46.2)	0.002^**^

^**†**^Median (25–75th percentile)

^*^represents *p*-value < 0.05

^**^represents *p*-value < 0.01

^***^represents *p*-value < 0.001

The majority of patients had no prior history of kidney comorbidities (89.9%). Three patients had chronic kidney disease (CKD) due to failing Fontan circulation, focal sclerosing glomerulonephritis, and lupus nephritis, respectively. The etiology of CKD in one patient was unknown. There were seven patients with kidney abnormalities, but no evidence of CKD at admission. These abnormalities included horseshoe kidneys, duplications of the collecting system, solitary kidneys, and post-infectious glomerulonephritis associated with rheumatic heart disease.

The failure group had larger proportions of patients with pre-existing cardiac disease (38.5% vs. 19.2%, *p* = 0.063) and patients who received cardiopulmonary bypass during their admission (15.4% vs. 5.5%, *p* = 0.201), although neither of these reached statistical significance.

Diagnostic groupings were not significantly different between the success and failure groups. The majority of patients were diagnosed with sepsis during their admission (54.5% overall, 57.5% in success group, 46.2% in failure group, *p* = 0.364). The failure group had a proportionally higher number of oncologic patients, although this association was not significant (30.8% vs. 13.7%, *p* = 0.075). The reasons for CKRT initiation were similar between the two groups. More patients in the success group received CKRT for sepsis than the failure group (28.8% vs. 15.4%, *p* = 0.202).

The median duration of the initial CKRT course was 171 h in the success group and 114 h in the failure group (*p* = 0.462). Both groups had similar numbers of patients who received CKRT while on ECMO (20.5% vs. 23.1% in the success and failure groups, respectively, *p* = 0.785). We evaluated patients for use of other modalities of intermittent kidney replacement therapy prior to CKRT, after CKRT, and on hospital discharge and found no significant differences between the two groups.

The failure group experienced longer ICU length of stay (median 44.5 days vs. 27.2 days, *p* = 0.046) and higher in-hospital mortality (46.2% vs. 15.1%, *p* = 0.002).

### CKRT course

At CKRT initiation, the success and failure groups were similar with regard to vasoactive requirements (Table 2), MAP, need for mechanical ventilation, and fenoldopam usage (Supplemental Table 1). CVP was comparable between the success and failure groups as well (minimum CVP 10 vs. 10 mmHg, *p* = 0.857; maximum CVP 15 vs. 17 mmHg, *p* = 0.287). Administration of diuretics was similar between both groups, both in terms of the number of patients receiving diuretics (Table 2) and diuretic dosage in FE/kg (Supplemental Table 1). There were no significant differences in pH, lactate, serum creatinine, and albumin; however, BUN levels were elevated in the failure group (66 vs. 49 mg/dL, *p* = 0.043). The white blood cell count, hemoglobin, platelet count, CRP, and serum electrolytes (potassium, bicarbonate, and phosphorus) were also similar (Supplemental Table 1). Finally, both groups had equivalent percentage fluid overload at CKRT initiation (12.3% vs. 12.7%, *p* = 0.712) and urine output in the 6 h prior to CKRT initiation (1.0 vs. 0.9 mL/kg/h, *p* = 0.619).

**Table 2:** Hemodynamic, clinical, and biochemical characteristics of patients at initiation and in the 6 and 24 h prior to discontinuation of continuous kidney replacement therapy

	CKRT outcome		
	Success *n* = 73	Failure *n* = 26	*p* value
**CKRT start characteristics**			
Vasoactive inotropic score†	8.5 (0.0–25.0)	10.5 (0.0–19.8)	0.977
Central venous pressure	*n* = 65	*n* = 23	
Minimum†	10 (6–14)	10 (7–13)	0.857
Maximum†	15 (12–19)	17 (14–21)	0.287
Mechanical ventilation, *n* (%)	63 (86.3)	23 (88.5)	1.000
Diuretic use, *n* (%)	30 (41.1)	15 (57.7)	0.172
pH†	7.34 (7.29–7.40)	7.36 (7.30–7.42)	0.666
Lactate (mmol/L)†	2.00 (1.55–4.65)‡	1.65 (1.08–3.75)	0.250
BUN (mg/dL)†	49 (24–78)	66 (43–90)	0.043^*^
Serum creatinine (mg/dL)†	1.60 (1.00–3.27)‡	1.63 (1.36–2.50)	0.675
Albumin (mg/dL)†‡	2.8 (2.6–3.5)	2.7 (2.5–3.0)	0.115
% Fluid overload†	12.3 (5.4–26.3)	12.7 (6.7–27.4)	0.712
6-h UO (mL/kg/h) †	1.0 (0.2–2.5)	0.9 (0.2–1.9)	0.619
**CKRT stop characteristics**			
Vasoactive inotropic score†	0.0 (0.0–6.0)	4.8 (0.0–9.5)	0.068
Central venous pressure			
Minimum† Maximum†	5 (3–7)‡ 9 (7–12)‡	6 (5–9) 12 (8–15)	0.028^*^ 0.009^**^
Mechanical ventilation, *n* (%)	58 (79.5)	23 (88.5)	0.386
Diuretic use, *n* (%)	36 (49.3)	10 (38.5)	0.369
pH†	7.42 (7.37–7.45)	7.39 (7.33–7.45)	0.180
Lactate (mmol/L)†‡	1.40 (1.10–1.95)	1.65 (1.20–2.10)	0.661
BUN (mg/dL)†	17 (12–27)	21 (13–34)	0.371
Serum creatinine (mg/dL)†	0.51 (0.31–0.72)	0.50 (0.32–0.72)	0.905
Albumin (mg/dL)†‡	3.3 (3.0–3.7)	3.2 (2.89–3.6)	0.539
% Fluid overload†	8.8 (3.4–21.2)	11.5 (4.3–34.1)	0.443
Change in % FO†	− 2.8 (− 7.9 to + 3.3)	− 0.6 (− 5.8 to + 5.9)	0.443
6-h UO (mL/kg/h) †	0.8 (0.1–2.4)	0.1 (0.0–0.5)	< 0.001^***^
UO with diuretics†	1.7 (0.7–2.9)	1.0 (0.2–1.5)	0.183
UO without diuretics†	0.4 (0.0–1.1)	0.0 (0.0–0.1)	0.002^**^
24-h UO (mL/kg/h) †	0.8 (0.2–2.1)	0.2 (0.0–0.6)	0.006^**^
UO without diuretics†	1.7 (0.5–2.7)	0.9 (0.2–2.4)	0.299
UO without diuretics†	0.3 (0.1–1.4)	0.1 (0.0–0.3)	0.025^*^

^**†**^Median (25–75th percentile)

^‡^Greater than 10% of patients without available data

^*^represents *p*-value < 0.05

^**^represents *p*-value < 0.01

^***^represents *p*-value < 0.001

Prior to CKRT discontinuation, we again identified no significant differences between the groups in VIS, MAP, mechanical ventilation requirement, diuretic use and FE/kg, and fenoldopam use (Table 2, Supplemental Table 1). Both minimum and maximum CVP were significant (5 vs. 6 mmHg, *p* = 0.028 and 9 vs. 12 mmHg, *p* = 0.009, respectively) with higher CVPs seen in the failure group. Neither the overall percentage fluid overload since admission until CKRT discontinuation (8.8% vs. 11.5%, *p* = 0.443) nor the change in percentage fluid overload during the initial CKRT course (− 2.8% vs. − 0.6%, p = 0.443) was found to be significant.

The 6-h and 24-h urine outputs prior to CKRT discontinuation were significant (Table 2). Urine output in the 6 h prior to CKRT discontinuation in the success group had a median of 0.8 mL/kg/h compared to 0.1 mL/kg/h in the failure group (*p* < 0.001). In the 24-h period, urine output for the success group was 0.8 mL/kg/h; the failure group had a urine output of 0.2 mL/kg/h (*p* = 0.006). When categorizing urine output based on diuretic use status, only urine output without diuretics remained significant in both time periods.

For the time periods evaluated after CKRT discontinuation, urine output remained significant irrespective of diuretic administration (Table 3). In the 6 h after CKRT discontinuation, the success group demonstrated a higher median urine output than the failure group (1.8 vs. 0.1 mL/kg/h, *p* = 0.001). Urine output for those who received diuretics remained significant (2.1 vs. 1.1 mL/kg/h, *p* = 0.030) as did urine output without diuretics (0.1 vs. 0.0 mL/kg/h, *p* = 0.024). In the 12 h after CKRT discontinuation, urine outputs for all patients (2.4 vs. 0.1 mL/kg/h, *p* < 0.001), for patients receiving diuretics (3.0 vs. 0.4 mL/kg/h, *p* < 0.001), and for patients not receiving diuretics (0.1 vs. 0.0 mL/kg/h, *p* = 0.005) were significant with higher urine outputs seen in the success group compared to the failure group.

**Table 3:** Predictors in the 6 and 12 h after cessation of continuous kidney replacement therapy

	CKRT outcome		
	Success *n* = 73	Failure *n* = 26	*p* value
**6 h post-CKRT characteristics**			
Vasoactive inotropic score†	0.0 (0.0–6.0)	3.4 (0.0–10.3)	0.110
Central venous pressure			
Minimum† Maximum†	5 (3–8)‡ 10 (8–12)‡	7 (5–10) 11 (9–21)	0.046^*^ 0.078
Diuretic use, *n* (%)	51 (69.9)	16 (61.5)	0.470
% Fluid overload†	0.9 (0.1–2.0)	1.4 (0.4–3.1)	0.191
6-h UO (mL/kg/h)†	1.8 (0.3–3.4)	0.1 (0.0–1.7)	0.001^**^
UO with diuretics†	2.1 (0.8–3.5)	1.1 (0.2–2.4)	0.030^*^
UO without diuretics†	0.1 (0.0–2.6)	0.0 (0.0–0.0)	0.024^*^
**12 h post-CKRT characteristics**			
Vasoactive inotropic score†	0.0 (0.0–6.0)	3.9 (0.0–11.8)	0.152
Central venous pressure			
Minimum† Maximum†	5 (2–7)‡ 11 (9–13)‡	5 (4–10) 15 (10–22)	0.074 0.013^*^
Diuretic use, *n* (%)	53 (72.6)	19 (73.1)	1.000
BUN (mg/dL)†	31 (17–41)‡	37 (23–58)	0.105
Serum creatinine (mg/dL)†	0.80 (0.44–1.31)‡	0.92 (0.61–1.26)	0.335
% Fluid overload†	1.3 (0.2–3.0)	2.5 (0.8–6.3)	0.033*
12-h UO (mL/kg/h)†	2.4 (0.5–3.9)	0.1 (0.0–1.8)	< 0.001^***^
UO with diuretics†	3.0 (1.0–3.9)	0.4 (0.0–2.0)	< 0.001^***^
UO without diuretics†	0.1 (0.0–3.2)	0.0 (0.0–0.0)	0.005^**^

^**†**^Median (25–75th percentile)

^‡^Greater than 10% of patients without available data

^*^represents *p*-value < 0.05

^**^represents *p*-value < 0.01

^***^represents *p*-value < 0.001

In the 6 and 12 h after CKRT discontinuation, the failure group demonstrated higher median CVP than the success group (7 mmHg vs. 5 mmHg in 6-h period, *p* = 0.046; 15 mmHg vs. 11 mmHg in 12-h period, *p* = 0.013). Percentage fluid overload in the 12-h period since CKRT discontinuation was significant with greater gain in net intake and output in the failure group compared to the success group (1.3% vs. 2.5%, *p* = 0.033). The remaining hemodynamic, clinical, and laboratory variables were not significant in our analysis.

**Fig. 2:**
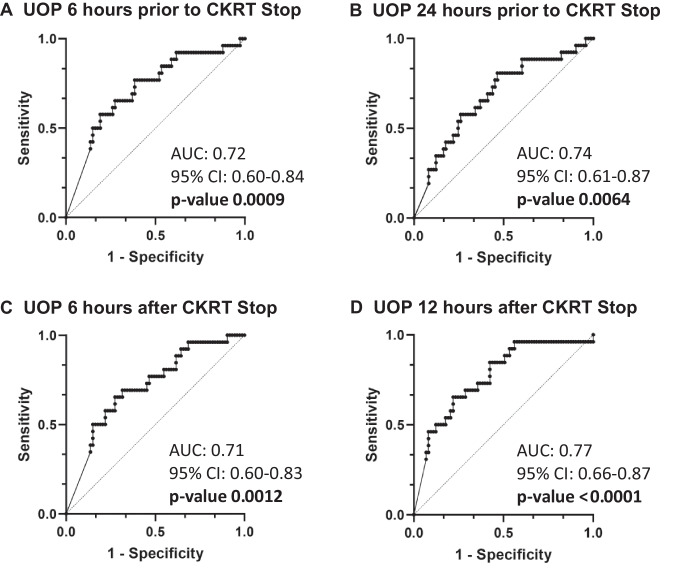
Receiver operating characteristic curves of urine output. **A** six hours and **B** 24 hours prior to cessation of continuous kidney replacement therapy. **C** six hours and **D** 12 hours after continuous kidney replacement therapy discontinuation

Each of the four ROC curves evaluating urine output in Fig. 2 had an area under the curve (AUC) greater than 0.7 (Fig. 2). The urine output threshold predictive of successful discontinuation in the 6 h prior to CKRT discontinuation was 0.51 mL/kg/h. This threshold demonstrated sensitivity and specificity of 0.616 and 0.769, respectively.

The multivariable logistic regression analysis did not identify any variables that were significant independent predictors of CKRT discontinuation (Table 4, all *p* values > 0.05). The 6-h and 24-h urine outputs prior to CKRT discontinuation demonstrated odds ratios of 1.366 (95% CI 0.937–1.992, *p* = 0.104) and 1.471 (95% CI 0.951–2.275, *p* = 0.083), respectively.

**Table 4:** Univariable and multivariable logistic regression analysis of factors associated with successful discontinuation of continuous kidney replacement therapy

	Univariable analysis			Multivariable analyses		
	Odds ratio	95% CI	*p* value	Odds ratio	95% CI	*p* value
CKRT duration	1.008	0.957, 1.070	0.785			
BUN at CKRT initiation	0.993	0.982, 1.004	0.207			
Cardiopulmonary bypass	3.136	0.691, 14.292	0.127			
CKRT initiation for sepsis	0.450	0.121, 1.352	0.185			
Pre-existing cardiac disease	2.634	0.975, 7.061	0.053	2.314	0.842, 6.302	0.100
6-h UO prior to CKRT discontinuation	1.402	0.965, 2.037	0.076	1.366	0.937, 1.992	0.104
24-h UO prior to CKRT discontinuation	1.487	0.971–2.277	0.068	1.471	0.951, 2.275	0.083

6 h and 24 h UO prior to CKRT discontinuation were analyzed in separate multivariable logistic regressions

## Discussion

In this retrospective cohort study, we sought to identify factors associated with successful CKRT discontinuation. The key finding of this study is that urine output greater than 0.5 mL/kg/h in the 6 h prior to CKRT discontinuation was predictive of successful CKRT withdrawal. Administration of diuretics decreased the strength of this association.

The KDIGO guidelines recommend discontinuation of kidney replacement therapy (KRT) “when it is no longer required, either because intrinsic kidney function has recovered to the point that it is adequate to meet patient needs, or because [RRT] is no longer consistent with the goals of care *(Not graded)*” [18]. The ambiguity of this recommendation reflects the lack of data regarding indicators of kidney recovery for patients receiving KRT. Still less is known regarding the transition from CKRT to intermittent kidney replacement therapy after a period of critical illness in patients with persistent kidney injury. Since the publication of the KDIGO guidelines in 2012, adult literature has begun to address this knowledge gap. In a multinational cohort analysis, Uchino et al. found that urine output in the 24 h prior to CKRT discontinuation was the strongest predictor of successful CKRT discontinuation [19]. The DoNE-RRT systematic review and meta-analysis also concluded that urine output was the most robust predictor of CKRT discontinuation in adult patients with a pooled sensitivity and specificity of 66.2% and 73.6%; however, given the heterogeneity of the studies, a threshold urine output could not be determined [20]. Our data supports the findings from adult literature and adds the first pediatric information regarding CKRT cessation.

The KDIGO guidelines provided strong recommendations against the use of diuretics “to enhance kidney function recovery, or to reduce the duration or frequency of RRT *(grade 2B)*” [18]. Despite this recommendation, clinical practice often incorporates loop diuretic use both while on CKRT and in the recovery phase after CKRT discontinuation. Similar to our data, Uchino et al. found that diuretic use during CKRT decreased the strength of the association between successful CKRT discontinuation and urine output [19]. Diuretic administration during CKRT was assessed in relation to fluid overload status in a recent study by Hall et al. [21]. Investigators found that diuretic use was not independently associated with the ability to achieve a set fluid balance goal while on CKRT. Together with our findings, these data suggest that administration of diuretics during CKRT may increase urine output but is unlikely to significantly improve fluid balance goals and does not predict intrinsic kidney recovery and ability to transition off CKRT.

As for the use of diuretics after CKRT discontinuation during the recovery phase of AKI, data is conflicting. In a retrospective study of 86 patients who underwent 101 CKRT weaning trials, Raurich et al. found that urine output in 6 h after CKRT cessation was a strong predictor of success and that furosemide use augmented this association [22]. Investigators found an AUC 0.94 (95% CI 0.88–1.0) in patients receiving furosemide and 0.85 (95% CI 0.72–0.99) in patients who did not. Similarly, Jeon et al. found on multivariable regression analysis that diuretic use (OR 5.529, 95% CI 4.120–7.410, *p* value = 0.001) and 24-h urine output prior to CKRT discontinuation (OR 1.001, 95% CI 1.001–1.002, *p* value < 0.001) were predictive factors of successful CKRT cessation in a retrospective cohort of 1176 patients [23]. However, a double blinded, randomized control trial assessing furosemide administration after CKRT discontinuation in adult ICU patients found no change in kidney failure duration and frequency of kidney recovery despite higher urine output and improved sodium excretion [24]. Our data supports the findings of the retrospective trials; diuretics did not detract from the strength of the association between urine output after CKRT discontinuation and sustained transition off CKRT.

Current practice guiding CKRT discontinuation assesses for the improvement or resolution of the underlying illness or insult contributing to AKI, evidence of kidney recovery via urine output, and appropriate fluid balance [25]. Surprisingly, variables related to underlying illness, namely, VIS, mechanical ventilation, oxygenation index, lactate, and CRP, were not significant between the success and failure groups. Furthermore, percentage fluid overload, which is a known independent predictor of mortality in critically ill patients, was not significant at CKRT initiation, discontinuation, or in the 6 h after CKRT discontinuation. Percentage FO at 12 h after CKRT discontinuation was significant but likely reflected low urine output in the failure cohort and the consequent inability to maintain adequate fluid balance.

From a practical standpoint, urine output in the 6-h period prior to CKRT discontinuation is a useful adjunct in guiding physicians in determining when a patient may successfully trial off CKRT. The 6-h period after CKRT discontinuation is also potentially useful, albeit predictors prior to cessation would be ideal [22]. CKRT filters currently require exchange every 72 h. Since the median duration of the initial CKRT course in our patients was 152 h, this suggests that patients underwent at least 2 circuit changes. These circuit changes provide an opportunity for clinicians to trial patients off CKRT while monitoring urine output, fluid status, and biochemical values.

Tourneur et al. compared protocolized CKRT weaning in adult patients with physician-directed CKRT discontinuation [26]. The investigators analyzed patients with AKI on CKRT transferred to two stepdown ICU units. For the patients in the unit randomized to the CKRT weaning protocol, patients were immediately trialed off CKRT for a period of 12 h. The trial off CKRT was aborted if patients remained anuric, had clinically relevant fluid overload, hyperkalemia, or hyper/hyponatremia, uremic complications, or persistent metabolic acidosis with pH < 7.1. During the trial off, patients maintained urine output of 0.5 mL/kg/h through furosemide infusion and the addition of hydrochlorothiazide as needed. Electrolyte abnormalities were managed conservatively, and hemodynamics (MAP > 65 and central venous oxygen saturation > 70%) were supported as needed with vasoactive medications, fluid resuscitation, or transfusion. This study found no difference in the number of patients who successfully weaned off CKRT (12 of 15 patients in each group); however, time from ICU admission to CKRT weaning was longer in the physician-directed group (median 5 days vs. 1.5 days).

Based on our findings, pediatric patients with urine output > 0.5 mL/kg/h while on CKRT should be considered for CKRT discontinuation. Patients who do not respond to diuretics during their trial off CKRT should be monitored closely as re-initiation of CKRT or other kidney replacement therapies may be warranted.

The strengths of our study include utilizing a substantial pediatric cohort. We did not exclude any patients, and thus, our data may be more generalizable. We also assessed not just clinical and biochemical variables at one time point but evaluated multiple time points during each patient’s CKRT course.

Despite our relatively large sample size, our study is still underpowered to allow appropriate assessment of subpopulations with increased risk of failure of CKRT weaning, namely, patients with sepsis, patients with cardiac disease who underwent cardiopulmonary bypass, neonatal patients, and patients who required CKRT through ECMO. To study these specific patients, future directions include conducting multicenter studies to determine predictors of kidney recovery on CKRT in these patients with AKI. Our small number of failure patients (*n* = 26) contributed to the lack of statistical power on multivariable logistic regression analysis to detect independent predictors of successful CKRT discontinuation. With a larger sample size, urine output in the periods surrounding CKRT discontinuation would likely have been an independent predictor of successful CKRT cessation given the strength of the univariable results.

In our study, patients who transitioned from CKRT to an intermittent KRT were not considered failures. Further subgroup analyses of patients requiring KRT after CKRT discontinuation emphasized the importance of urine output (Supplemental Table 2). Compared with the patients who did not require further kidney replacement therapies (KRT), patients requiring KRT more often required CKRT initiation for anuria/oliguria, uremia, and/or electrolyte abnormalities. They also demonstrated persistent oliguria (urine output < 0.5 mL/kg/h) at all-time points, i.e., prior to CKRT initiation, in the 6- and 24-h periods prior to discontinuation, and in the 6- and 12-h periods after CKRT cessation. Patients with complete kidney recovery were more likely to have CKRT initiation for sepsis, acidosis, and hyperammonemia and demonstrated median urine outputs > 1 mL/kg/h at all-time points. Future studies are needed to delineate predictors of CKRT discontinuation due to kidney recovery from predictors of successful transition to less resource-intensive kidney replacement therapies.

Serum creatinine as a marker of AKI has been repeatedly shown to be lacking in sensitivity and specificity. More emphasis has been placed on novel biomarkers, such as NGAL and cystatin C. Our study did not include these biochemical markers given the inconsistency in which it was obtained in our patient population across the study period. A future cohort study should obtain measurements of these biomarkers given the literature showing their correlation with the development of AKI and with severity of kidney injury. Ultimately, a multivariable model may be the best predictive tool to guide CKRT discontinuation.

## Conclusion

We report the first study assessing predictors of CKRT termination in pediatric patients. Urine output of at least 0.5 mL/kg/h in the 6 h prior to CKRT discontinuation was most predictive of successful discontinuation. Future prospective multiinstitutional studies are necessary to validate this urine output threshold and to assess novel biomarkers of kidney injury and recovery. Continued research into optimal timing of CKRT discontinuation is vital to preventing CKRT complications, reducing length of stay, and promoting kidney recovery.

## Supplementary Information

Below is the link to the electronic supplementary material.

Graphical Abstract (PPTX 256 KB)

Supplementary file2 (DOCX 22 KB)

Supplementary file3 (DOCX 31 KB)
