# Supplementary material for: Predictors of successful discontinuation of continuous kidney replacement therapy in a pediatric cohort
Source: Pediatr Nephrol. 2022 Oct 31;38(7):2221–31. doi: 10.1007/s00467-022-05782-0 (PMC10234862; doi:10.1007/s00467-022-05782-0)
Supplement: Supplementary file 1 — Graphical Abstract (PPTX 256 KB) [file 467_2022_5782_MOESM1_ESM.pptx]

## Slide 1
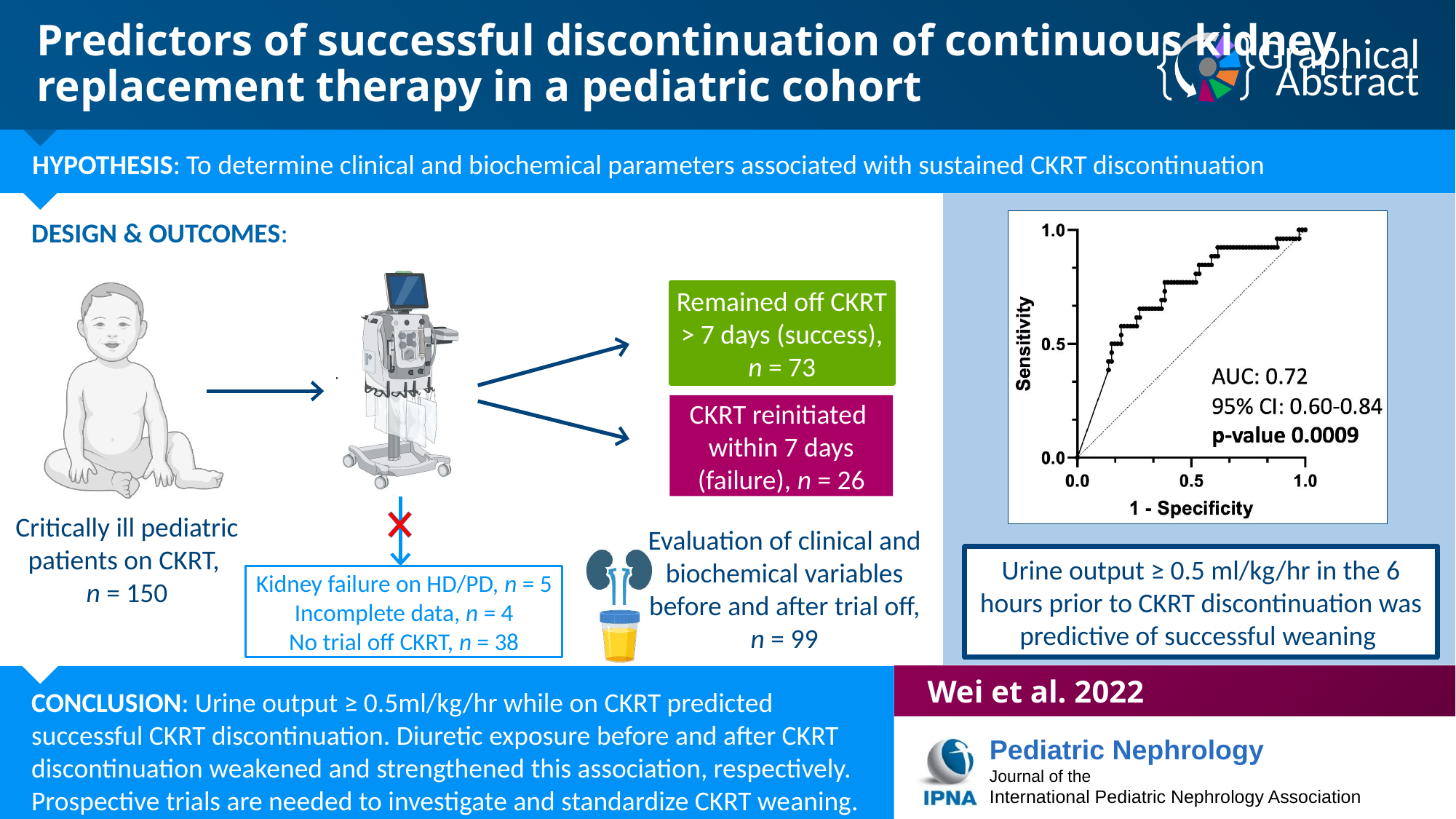

Predictors of successful discontinuation of continuous kidney
replacement therapy in a pediatric cohort
HYPOTHESIS: To determine clinical and biochemical parameters associated with sustained CKRT discontinuation
DESIGN & OUTCOMES:
Remained off CKRT > 7 days (success), n = 73
CKRT reinitiated
within 7 days (failure), n = 26
Critically ill pediatric patients on CKRT,
n = 150
Evaluation of clinical and biochemical variables before and after trial off,
n = 99
Urine output ≥ 0.5 ml/kg/hr in the 6 hours prior to CKRT discontinuation was predictive of successful weaning
Kidney failure on HD/PD, n = 5
Incomplete data, n = 4
No trial off CKRT, n = 38
Wei et al. 2022
CONCLUSION: Urine output ≥ 0.5ml/kg/hr while on CKRT predicted successful CKRT discontinuation. Diuretic exposure before and after CKRT discontinuation weakened and strengthened this association, respectively. Prospective trials are needed to investigate and standardize CKRT weaning.
