# Supplementary material for: Predictors of successful discontinuation of continuous kidney replacement therapy in a pediatric cohort
Source: Pediatr Nephrol. 2022 Oct 31;38(7):2221–31. doi: 10.1007/s00467-022-05782-0 (PMC10234862; doi:10.1007/s00467-022-05782-0)
Supplement: Supplementary file 2 — Supplementary file2 (DOCX 22 KB) [file 467_2022_5782_MOESM2_ESM.docx]

**Supplemental Table 1** Additional hemodynamic, clinical and biochemical variables at initiation, discontinuation and after discontinuation in patients trialed off continuous renal replacement therapy

|  | CKRT Outcome | |  |
| --- | --- | --- | --- |
|  | Success  *n* = 73 | Failure  *n* = 26 | p-value |
| CKRT Start Characteristics |  |  |  |
| Mean arterial pressure (mmHg)  Minimum†  Maximum† | 66 (54-74)  88 (73-97) | 67 (53-79)  93 (76-100) | 0.808  0.548 |
| Furosemide equivalents/kg† | 0.0 (0.0-1.5) | 0.4 (0.0-2.6) | 0.186 |
| Fenoldopam, n (%) | 20 (27.4) | 8 (30.8) | 0.802 |
| Potassium (mEq/L)† | 4.0 (3.4-4.7) | 3.8 (3.1-4.4) | 0.138 |
| Bicarbonate (mEq/L)† | 24.00 (19.00-27.00) | 24.50 (21.00-26.00) | 0.551 |
| Phosphorus (mmol/L)†‡ | 5.60 (4.32-6.60) | 6.40 (5.00-7.00) | 0.196 |
| CRP (mg/dL)†‡ | 7.40 (2.80-20.30) | 6.00 (4.60-17.38) | 0.599 |
| CKRT Stop Characteristics |  |  |  |
| Mean arterial pressure (mmHg)  Minimum†  Maximum† | 68 (57-79)  90 (75-100) | 63 (52-73)  83 (72-103) | 0.219  0.548 |
| Furosemide equivalents/kg† | 0.0 (0.0-1.8) | 0.0 (0.0-3.5) | 0.876 |
| Fenoldopam, n (%) | 11 (15.1) | 5 (19.2) | 0.757 |
| Potassium (mEq/L)† | 3.7 (3.4-4.2) | 3.6 (3.2-4.2) | 0.511 |
| Bicarbonate (mEq/L)† | 29 (27-32) | 28 (24-30) | 0.036* |
| Phosphorus (mmol/L)† | 4.4 (3.8-4.7) | 4.0 (2.5-4.5) | 0.079 |
| 6 hours post CKRT discontinuation |  |  |  |
| Mean arterial pressure (mmHg)  Minimum†  Maximum† | 70 (58-83)  90 (77-104) | 63 (53-74)  85 (72-98) | 0.106  0.304 |
| Furosemide equivalents/kg† | 0.7 (0.0-2.8) | 0.8 (0.0-4.4) | 0.871 |
| Fenoldopam, n (%) | 10 (13.7) | 5 (19.2) | 0.531 |
| 12 hours post CKRT discontinuation |  |  |  |
| Mean arterial pressure (mmHg)  Minimum†  Maximum† | 67 (55-79)  94 (82-107) | 58 (53-74)  90 (78-102) | 0.129  0.456 |
| Furosemide equivalents/kg† | 1.3 (0.0-4.0) | 2.1 (0.1-6.2) | 0.389 |
| Fenoldopam, n (%) | 10 (13.7) | 4 (16.0) | 0.749 |
| Potassium (mEq/L)† | 3.7 (3.4-4.2)‡ | 3.6 (3.3-4.1) | 0.614 |
| Bicarbonate (mEq/L)† | 28 (26-31)‡ | 27 (24 -29) | 0.087 |
| Phosphorus (mmol/L)†‡ | 4.7 (4.0-5.5) | 4.0 (3.3-5.5) | 0.093 |

† Median (25^th^ – 75^th^ percentile)

‡ Greater than 10% of patients without available data
