# Supplementary material for: Predictors of successful discontinuation of continuous kidney replacement therapy in a pediatric cohort
Source: Pediatr Nephrol. 2022 Oct 31;38(7):2221–31. doi: 10.1007/s00467-022-05782-0 (PMC10234862; doi:10.1007/s00467-022-05782-0)
Supplement: Supplementary file 3 — Supplementary file3 (DOCX 31 KB) [file 467_2022_5782_MOESM3_ESM.docx]

**Supplemental Table 2** Characteristics of patients requiring additional dialytic therapy following successful trial off CKRT

|  | KRT Requirement Following CKRT Discontinuation | |  |
| --- | --- | --- | --- |
|  | No KRT Required  *n* = 41 | KRT Required  *n* = 32 | p-value |
| Age (year)† | 4.5 (0.8-9.2) | 11.0 (2.7-14.7) | 0.054 |
| Female, n (%) | 22 (53.7) | 15 | 1.000 |
| Admission weight (kilograms)† | 18 (7.2-31.2) | 32.5 (12.4-54.3) | 0.145 |
| Pre-existing kidney disease |  |  |  |
| None, n (%) | 39 (95.1) | 29 (90.6) | 0.893 |
| CKD, n (%) | 0 (0) | 2 (6.3) | 0.441 |
| Other, n (%) | 2 (4.9) | 1 (3.1) | 1.000 |
| Pre-morbid conditions |  |  |  |
| Cardiac disease, n (%) | 7 (17.1) | 7 (21.9) | 0.766 |
| Liver disease, n (%) | 4 (9.8) | 4 (12.5) | 1.000 |
| PRISM III Score | 12 (6-22) | 12.5 (11-18) | 1.000 |
| Diagnostic Group |  |  |  |
| Post-operative, n (%) | 4 (9.8) | 4 (12.5) | 1.000 |
| Post-op cardiopulmonary bypass, n (%) | 2 (4.9) | 2 (6.3) | 1.000 |
| Cardiovascular, n (%) | 11 (26.8) | 8 (25.0) | 1.000 |
| Respiratory, n (%) | 9 (22.0) | 8 (25.0) | 1.000 |
| Neurologic, n (%) | 7 (17.1) | 5 (15.6) | 1.000 |
| Gastrointestinal, n (%) | 7 (17.1) | 8 (25.0) | 1.000 |
| Sepsis, n (%) | 22 (53.7) | 20 (62.5) | 0.966 |
| Oncologic, n (%) | 5 (12.2) | 5 (15.6) | 0.740 |
| Other, n (%) | 7 (17.1) | 6 (18.8) | 1.000 |
| Reason for CKRT Initiation |  |  |  |
| Fluid overload, n (%) | 26 (63.4) | 21 (65.6) | 1.000 |
| Anuria/oliguria, n (%) | 12 (29.3) | 24 (75.0) | <0.001*** |
| Electrolyte abnormality, n (%) | 4 (9.8) | 9 (29.0) | 0.183 |
| Uremia, n (%) | 5 (12.2) | 12 (37.5) | 0.042* |
| Acidosis, n (%) | 13 (31.7) | 6 (18.8) | 0.569 |
| Sepsis, n (%) | 17 (41.5) | 5 (15.6) | 0.113 |
| Hyperammonemia, n (%) | 6 (14.6) | 0 (0.0) | 0.095 |
| Other, n (%) | 1 (2.4) | 7 (21.9) | 0.054 |
| CKRT duration (hours)† | 91 (46-234) | 199 (104-274) | 0.081 |
| CKRT on ECMO, n (%) | 12 (29.3) | 3 (9.4) | 0.134 |
| ICU length of stay (days)† | 30.2 (11.7-57.7) | 23.6 (14.0-53.2) | 0.607 |
| Hospital length of stay (days)† | 45 (24.0-79.0) | 35 (24.8-65.8) | 0.950 |
| In-hospital mortality, n (%) | 8 (19.5) | 3 (9.4) | 0.328 |

† Median (25^th^ – 75^th^ percentile)

|  | CKRT Outcome | |  |
| --- | --- | --- | --- |
|  | No KRT Required  *n* = 41 | KRT Required  *n* = 32 | p-value |
| CKRT Start Characteristics | | | |
| Vasoactive inotropic score† | 11 (2.5-27) | 2.5 (0-19.8) | 0.220 |
| Central venous pressure  Minimum†  Maximum† | 10 (6-14.5)  16 (12-21.5) | 9.5 (5-12)  13 (11-18) | 1.000  0.268 |
| Mechanical ventilation, n (%) | 37 (90.2) | 26 (81.2) | 0.952 |
| Diuretic use, n (%) | 18 (43.9) | 12 (37.5) | 0.643 |
| pH† | 7.38 (7.31-7.43) | 7.324 (7.27-7.37) | 0.064 |
| Lactate (mmol/L)† | 2.3 (1.8-7.3) | 1.65 (1.0-1.9) | 0.003** |
| BUN (mg/dL)† | 30 (20-59) | 66.5 (47.5, 91.3) | 0.001** |
| Serum creatinine (mg/dL)† | 1.04 (0.65-1.43) | 3.47 (1.75-4.65) | <0.001*** |
| Albumin (mg/dL)† | 3.1 (2.5-3.5) | 2.8 (2.7-3.1) | 0.531 |
| % Fluid overload† | 15.1 (7.9-28.7) | 9.6 (3.3-21.4) | 0.144 |
| 6hr UO (mL/kg/hr) † | 1.92 (0.86-3.2) | 0.3 (0.1-1.0) | <0.001*** |
| CKRT Stop and Post Discontinuation Characteristics | | | |
| Vasoactive inotropic score† | 4 (0-8) | 0 (0-2) | 0.008** |
| Central venous pressure  Minimum†  Maximum† | 5 (4-7)  9 (7-12) | 4.5 (2.3-6)  8.5 (7-10.8) | 0.487  0.404 |
| Mechanical ventilation, n (%) | 35 (85.4) | 23 (71.9) | 0.579 |
| Diuretic use, n (%) | 15 (36.6) | 11 (34.4) | 0.102 |
| pH† | 7.41 (7.36-7.44) | 7.44 (7.40-7.46) | 0.140 |
| Lactate (mmol/L)†‡ | 1.7 (1.2-2.2) | 1.3 (1.1-1.4) | 0.131 |
| BUN (mg/dL)† | 17 (10.5-25.5) | 18 (14.5-29) | 0.449 |
| Serum creatinine (mg/dL)† | 0.39 (0.24-0.53) | 0.7 (0.56-1.26) | <0.001*** |
| Albumin (mg/dL)† | 3.4 (2.9-3.7) | 3.2 (3.1-3.7) | 1.000 |
| % Fluid overload† | 13.4 (3.9-30.9) | 6.5 (1.8-13.9) | 0.116 |
| Change in % FO† | -2.8 (-7.7-3.3) | -2.4 (-8.3-3.8) | 1.000 |
| 6hr UO (mL/kg/hr) † | 1.8 (0.8-2.9) | 0.1 (0-0.6) | <0.001*** |
| UO with diuretics† | 2.4 (1.2-3.7) | 0.6 (0.2-1.2) | 0.016* |
| UO without diuretics† | 1.3 (0.4-2.5) | 0.1 (0-0.4) | <0.001*** |
| 24hr UO (mL/kg/hr) † | 1.9 (0.8-2.9) | 0.1 (0.0-0.5) | <0.001*** |
| UO with diuretics† | 2.1 (0.9-3.1) | 0.4 (0.2-1.2) | 0.006* |
| UO without diuretics† | 1.5 (0.7-2.2) | 0.1 (0.0-0.3) | <0.001*** |
| 6hr UO (mL/kg/hr)† | 3.3 (1.8-5.7) | 0.3 (0.0-0.8) | <0.001*** |
| UO with diuretics† | 3.0 (1.8-5.1) | 0.7 (0.3-2.1) | 0.001** |
| UO without diuretics† | 5.8 (3.6-8.0) | 0.0 (0.0-0.1) | <0.001*** |
| 12hr UO (mL/kg/hr)† | 3.7 (3.0-4.6) | 0.4 (0.0-1.0) | <0.001*** |
| UO with diuretics† | 3.4 (2.6-4.5) | 0.7 (0.4-1.8) | <0.001*** |
| UO without diuretics† | 4.2 (3.8-5.7) | 0.0 (0.0-0.1) | 0.002** |

**†** Median (25^th^ – 75^th^ percentile)

‡ Greater than 10% of patients without available data
